# Enhanced Autolysosomal Function Ameliorates the Inflammatory Response Mediated by the NLRP3 Inflammasome in Alzheimer’s Disease

**DOI:** 10.3389/fnagi.2021.629891

**Published:** 2021-02-23

**Authors:** Wen Zhou, Deng Xiao, Yueyang Zhao, Botao Tan, Zhimin Long, Lehua Yu, Guiqiong He

**Affiliations:** ^1^Department of Neurorehabilitation, The Affiliated Rehabilitation Hospital of Chongqing Medical University, Chongqing, China; ^2^Department of Anatomy, Chongqing Medical University, Chongqing, China; ^3^Department of Rehabilitation Medicine, The Second Affiliated Hospital of Chongqing Medical University, Chongqing, China; ^4^Institute of Neuroscience, Chongqing Medical University, Chongqing, China

**Keywords:** Alzheimer’s disease, NLRP3 inflammasome, autophagy, TFEB, transgenic mice

## Abstract

The pathogenesis of Alzheimer’s disease (AD) involves activation of many NLRP3 inflammatory bodies, which may be related to amyloid β peptide and aggregation of misfolded proteins. Autophagy is an important regulator of inflammatory bodies. However, autophagy shows dynamic changes in the development of AD, and its role in inflammation remains controversial. In this study, the key link between autophagic disorders and the NLRP3 inflammasome in AD was investigated. APP/PS1 double transgenic mice and C57 mice with Aβ_25–35_ injected into the lateral ventricle were used as two animal models of AD. Immunofluorescence staining and Western blot analysis showed that NLRP3 inflammasome-related proteins and inflammatory cytokines, such as IL-1α, IL-1β, IL-6, IL-12, and TNF-α, were increased and microglia were activated in the brains of both AD animal models. Endogenous overexpression of the APPswe gene and exogenous addition of Aβ_25–35_ increased the expression of NLRP3 inflammasome-related proteins, while exogenous Aβ_25–35_ intervention more significantly activated inflammation. Furthermore, LC3 was increased in the AD animal and cell models, and the level of Lamp1 decreased. After overexpression of the primary regulator of lysosomal biogenesis, TFEB, the lysosome protein Lamp1 was increased, and LC3 and inflammatory protein expression were decreased. These results suggest that the NLRP3 inflammasome-mediated inflammatory response is activated in AD animal and cell models, which may be related to the decline in autolysosome function. Overexpression of the TFEB protein can reduce the inflammatory response by improving autolysosome function in AD model cells.

## Introduction

Alzheimer’s disease (AD), the most common type of dementia, is a neurodegenerative disease with cognitive impairment as the primary clinical manifestation (Ahmed et al., 2017; Teimouri et al., 2020). Central inflammatory reactions are involved in the pathological process of AD, and microglia and many molecular mediators participate in the inflammatory response (François et al., 2014). Glial cells aggregate near senile plaques in the brains of AD patients and experimental rats (Son et al., 2012), and these structures may be related to the activation of inflammatory bodies by aggregating amyloid proteins and misfolded proteins (Masters and O’Neill, 2011). The inflammatory response, with the NLRP3 inflammasome as the core, is an important part of the neuroimmune response (You et al., 2017; La Rosa et al., 2019). Studies have found that high levels of toxic Aβ aggregation may induce the activation of the NLRP3 inflammasome, mediate harmful chronic inflammatory reactions, and promote the progression of AD (François et al., 2014).

Autophagy, considered the main regulator of inflammatory bodies (Sun et al., 2017), is an important mechanism to inhibit the Aβ-induced neuroinflammatory response (Zhang et al., 2016). Autophagy plays multiple roles in regulating inflammatory cell activation and controlling the production, processing, and secretion of IL-1 family cytokines (Harris et al., 2017). Autophagy also prevents tissue inflammation by scavenging apoptotic bodies (BostancıKlıoğlu, 2019).

Unobstructed autophagic flux consists of the following steps: the formation of an autophagic initiator, the elongation and closure of the autophagic membrane, and the fusion and degradation of autophagic and lysosomal substrates (Kim et al., 2016). However, autophagy is usually disordered in AD (Peric and Annaert, 2015). And this dysfunction may occur in different stages of AD: autophagic activation, autophagic formation, and autophagy-lysosomal degradation (Zhang et al., 2016). Some studies have indicated that there may be excessive activation of autophagy in AD, which accelerates APP metabolism, resulting in a high level of Aβ production and aggravating this condition (Nixon et al., 2005). Other studies have also suggested that the autophagic flux of AD is damaged during the proteolytic stage of autolysosomes (Ahmed et al., 2017). The degradation function of lysosomes is decreased (Boland et al., 2008), and the degradation of autolysosomes is damaged, which may interfere with the processing of APP and cause pathological changes in AD (Salminen et al., 2013).

Many studies have shown that autophagy can negatively regulate the activation of the NLRP3 inflammasome, and the NLRP3 inflammasome can reverse the effect of autophagy (Levine et al., 2011). A deeper understanding of the mechanisms of the autophagic disorder and the interaction between neuroinflammation and autophagy in the pathogenesis of AD is essential for the development of novel therapeutic strategies for AD (BostancıKlıoğlu, 2019).

In this study, the NLRP3 inflammasome was observed *in vivo* and *in vitro* in an AD model. The results showed that immune-inflammatory activation occurred in AD animal and cell models and was mediated by the NLRP3 inflammasome. Autophagic flow detection in AD model mice and cells showed that autophagic degradation was abnormal. The decrease in lysosomal number or function may be the key factor in autophagic disorder. Through overexpression of TFEB, a major regulator of lysosome biogenesis, in cocultured cells, we detected the effects of TFEB on NLRP3 inflammatory bodies. We found that overexpression of the TFEB protein could increase the lysosomal-associated membrane protein Lamp1 and reduce the immune-inflammatory response in an AD cell model. In conclusion, the results of this study suggest that the NLRP3 inflammasome is activated to different degrees in AD animal models and cell models, which may be related to dysfunction of autophagic degradation caused by the decline in lysosomal function. Overexpression of the TFEB protein improves the function of autophagy-lysosomes, which may be attributed to the improvement in autophagic degradation, thus reducing the inflammatory response of AD cell models.

## Materials and Methods

### Preparation of Oligomeric Aβ_25–35_ Solution

Aβ_25–35_ (A4559, Sigma–Aldrich, St. Louis, MI, USA) was dissolved in sterilized double-distilled water, diluted in 1 mM/l mother liquor, aged in a 37°C water bath for 7 days, and then stored at −20°C for preservation (Pike et al., 1995).

### Animals

APP/PS1 double transgenic mice were purchased from the Institute of Laboratory Animal Sciences, Chinese Academy of Medical Sciences (Beijing, China). These APPswe/PS1-dE9 mice contain the mutation of mouse/human Swedish and human PS1-dE9 genes (Jankowsky et al., 2001; Wilcock and Colton, 2008; Esquerda-Canals et al., 2017). Wild-type C57 mice were obtained from the animal experiment center of Chongqing Medical University. All mice were male.They were reared in the animal feeding center of Chongqing Medical University. The feeding conditions were as follows: a 12-h light/dark cycle at 22°C standard conditions, a temperature of 23 ± 3°C, relative humidity of 55 ± 5% and circulating food and water available *ad libitum*.

The mice were divided into three groups: the WT (wild-type) group (*n* = 10), the APP/PS1 group (*n* = 10), and the Aβ_25–35_ group (*n* = 10). The C57 mice in the Aβ_25–35_ group were injected with Aβ_25–35_ in the lateral ventricle when they were 6 months old. The mice were anesthetized by intraperitoneal injection of 3% chloral hydrate at a rate of 0.1 ml/g. The mice were fixed on the brain localizer, the brain’s skin was cut, and a drop of H_2_O_2_ was added. After reaction for 30–60 s, the meninges were lifted and cut, and the sutures were exposed. The lateral ventricle (0.22 mm behind the front brain, 1.0 mm in the left and right sides, and 2.5 mm in depth) was located by a stereotactic instrument. Aβ_25–35_ solution (10 μg) was injected into the ventricle (Li et al., 2017). The injection rate was 1 μl/min. Each side was injected for 10 min, and the needle was withdrawn slowly after 5 min. The mice in the wild-type group were injected with Aβ_25–35_ diluent in the same way.

Animal care and laboratory procedures complied with the National Institute of Research Laboratory Care and Use of Health Guidelines for Animals and Chongqing Medical University animal use policy.

### Behavioral Tests

When the mice were 7 months of age, the Morris water maze (MWM) test was utilized to test the spatial cognitive function and memory of the mice. The circular pool (50 cm high, 2 cm in diameter, and 30 cm in depth) in the water maze was divided into four quadrants, and the escape platform was placed in the first quadrant. The pool’s water temperature was approximately 23°C, and white nontoxic dye was well mixed. In the visual platform experiment, the platform in the water tank came out of the water. Mice were placed in the water in four quadrants with an interval of 15 min. If the mice swam to the platform by themselves within 1 min, they were allowed to rest on the platform for 5 s; if the mice did not find the platform at the end of 1 min, they would rest on the platform for 20 s. The visual platform experiment lasted for 1 day. In the hidden platform experiment, the escape platform was placed 1 cm below the water surface, and the mice were placed into the water tank in four quadrants. The swimming rules were the same as those on the first day of the visual platform experiment. The hidden platform experiment lasted for 5 days. A computer video capture system recorded the swimming speed, path, and latency of the mice. The platform was removed on the sixth day, and the mice were placed in the pool at the third quadrant. The total time of each mouse in the pool was 60 s. The computer video system captures the time, path, and crossing times of mice to the platform point.

### Tissue Preparation

The mice were killed after the MWM test. The mice were deeply anesthetized with pentobarbital at 50 mg/kg. Normal saline (0.9%) was perfused through the heart to wash the cerebral circulation blood. Then, the brain tissue was quickly removed on a cold plate. Brain tissues that needed immunofluorescence analysis were fixed in 4% paraformaldehyde for 24 h and then dehydrated in 30% sucrose for 48 h. The treated brain tissue was fixed with an embedding agent and then cut into 3–5 μm thick slices on a freezing microtome.

### Inflammatory Factors Assay

Brain tissue samples were lysed with RIPA (P0013B, Beyotime, China) lysis buffer, and protein concentrations were determined using the BCA kit (P0010, Beyotime, China). The inflammatory factors in the samples were measured by the Bio-Plex Pro Mouse Cytokine Grp Panel 23-plex kit (#M60009RDPD, Bio-Rad, Hercules, CA, USA) according to the manufacturer’s instructions. Inflammatory factors concentrations were determined in the Bio-Plex MAGPIX System (Bio-Rad, Hercules, CA, USA).

### Cell Cultures and Treatments

SH-SY5Y cells transfected and stably expressing Swedish mutant APP (APPswe) and SH-SY5Y cells transfected with empty vector (APP_WT_) were donated by Professor Weihong Song at the University of British Columbia. BV2 cells were donated by Professor Zhifang Dong at the Children’s Hospital of Chongqing Medical University. The cell culture medium was composed of 10% DMEM (8119211, Gibco, USA), 90% FBS (c10310-1, Biological Industries, Israel), and double antibiotic consisting of 1% penicillin and streptomycin (c0222, Beyotime, China). The cells were cultured in a cell incubator containing 5% CO_2_ humidified at 37°C.

In this study, BV2 and neural cell coculture systems were used in all cell experiments *in vitro*. BV2 cells were seeded (5 × 10^5^ cells per well) in the lower layer of six-well plates (723101, NEST, China). After precipitation for 30 min, APPswe cells (2.5 × 10^5^ cells per well) or Aβ_25–35_-supplemented SH-SY5Y cells (2.5 × 10^5^ cells per well) were plated on the upper layer of the Transwell (Zujovic and Taupin, 2003; Chan et al., 2019; Lawrimore et al., 2019). Then, the cells were cocultured for 24 h for follow-up experiments.

In the LC3 turnover test, 10 nm bafilomycin A1 (#54645S, Sigma–Aldrich, St. Louis, MI, USA) was added to the SH-SY5Y control group or Aβ_25–35_ group (Aβ_25–35_ was added to SH-SY5Y cells) that were cocultured with BV2 cells. LC3 protein was detected by Western blot analysis after 2 h of the intervention (Chu, 2010; Klionsky et al., 2016; Yoshii and Mizushima, 2017).

SH-SY5Y cells transfected with empty vector were divided into APP_WT_ group, and SH-SY5Y cells transfected and stably expressing Swedish mutant APP were divided into APPswe group. After BV2 cells were cocultured with Aβ_25–35_-supplemented SH-SY5Y cells or APPswe cells, SH-SY5Y or APPswe cells were removed for analysis of autophagic protein expression. When the expression of inflammatory proteins in BV2 cells was detected, BV2 cells cocultured with APP_WT_ group cells were classified as the BV2_WT_ group, BV2 cells from cells cocultured with APPswe group cells were classified as the BV2_APP_ group, and BV2 cells cocultured with SH-SY5Y cells were divided into the BV_con_ (BV2 cocultured with SH-SY5Y cells no Aβ_25–35_) group or the BV2_Aβ_ (BV2 cocultured with SH-SY5Y cells treated by Aβ_25–35_) group.

For overexpression of TFEB, adenovirus (3 MOI, L/NAP20082408, Hanbio, China) or empty vector virus (3 MOI, L/NAP20083110, Hanbio, China) was added to SH-SY5Y cells treated with Aβ_25–35_, which were cocultured with BV2 cells (Yamamoto et al., 2019). After 24 h of infection, SH-SY5Y cells or BV2 cells were removed to detect protein expression. SH-SY5Y cells were tested in the Aβ group (SH-SY5Y treated with Aβ_25–35_), the Aβ + EV (empty virus) group, and the Aβ + TFEB (overexpression of TFEB) group. For detection of BV2 cells, the groups were BV2_Aβ_ group (BV2 cocultured with SH-SY5Y cells treated with Aβ_25–35_), BV2_Aβ_ + EV (empty virus) group, and BV2_Aβ_ + TFEB (overexpression of TFEB) group.

### EdU Assays

SH-SY5Y cells were cultured in 24-well plates and treated with 10, 20, or 40 μM Aβ_25–35_ for 24 h, and sterilized double-distilled water was added to the control group. After the intervention, 50 μm EdU reagent (c10310-1, RiboBio, China) was added to SH-SY5Y cells to label the nuclei, and then, the cells were incubated in a 37°C incubator for 2 h. After the intervention, the cells were fixed according to the kit’s instructions and stained with Apollo for 30 min and DAPI for 5 min. After cell samples were prepared, images were taken by a fluorescence microscope. Blue staining was used for all nuclei, and red staining was used for proliferating nuclei. The proportion of red nuclei to blue nuclei in each field was calculated and was the cell proliferation rate.

### Western Blot

The cells were washed twice with cold PBS buffer and lysed on ice with RIPA (P0013B, Beyotime, China) lysis buffer for 30 min. The lysate was centrifuged at 4°C for 15 min at 14,000 *g*. The supernatant was collected, and protein was extracted by the Beyotime BCA kit (P0010, Beyotime, China) to determine the protein concentration. The protein was then denatured in loading buffer and denatured by boiling in a 95°C water bath for 5 min. The protein samples (20 μg of protein per gel lane) were loaded onto SDS–PAGE gels and then transferred to PVDF membranes. The membranes were blocked in 5% bovine serum albumin (BSA) for 2 h at room temperature and incubated overnight in the primary antibody at 4°C. The antibody information is as follows: LC3 (#3868 s, Cell Signaling Technology, USA), Lamp1 (ab24170, Abcam, UK), NLRP3 (bs-10021R, Bioss, China), Caspase-1 (#3866, Cell Signaling Technology, USA), Caspase-1 (bs-0169R, Bioss, China), IL-1β (bs-0812R, Bioss, China), TFEB (ab270604, Abcam, UK), and β-actin (AA128-1, Beyotime, China). The PVDF membrane was washed and incubated with horseradish peroxidase and secondary antibody for 1 h. An enhanced chemiluminescence kit (BL520B, Biosharp, China) and gel imaging system (Bio-Rad, USA) were utilized to collect protein development images. ImageJ software was used for the quantitative analysis of signals.

### Real-Time Quantitative RT-PCR

TRIzol reagent (B511321, Sangon Biotech, China) was used to extract total RNA from each group of cells. Random hexamer primers and MMLV reverse transcriptase (m5301, Promega, USA) were used to reverse transcribe cDNA. SGExcel FastSYBR Mixture (B532954, Sangon Biotech, China) was utilized for quantitative PCR, and Stratagene mx3000p (Agilent Technology) was used for gene transcription quantification. The primers for detecting Lamp1 and β-actin mRNA were synthesized by Sangon Biotech Co., Limited (Shanghai, China), and the sequences were as follows: Lamp1: forward, CTCTGTGGACAAGTACAACGT and reverse, GTTGATGTTGAGAAGCCTTGTC; β4-actin: forward, TCGTGCGTGACATCAAAGAC and reverse, CAAGAAGGAAGGCTGGAAAA.

### Immunofluorescence

Brain tissue samples were washed with PBS three times and sealed with 5% BSA blocking solution at room temperature for 1 h. The corresponding primary antibody was selected to incubate the sectioned tissue, and the antibody information is as follows: LC3 (GB13431, Servicebio, China), Lamp1 (bs-1970R, Bioss, China), NLRP3 (GB11300, Servicebio, China), Caspase-1 (GB11383, Servicebio, China), IL-1β (GB11113, Servicebio, China), IBA-1 (GB12105, Servicebio, China), and iNOS (GB11119, Servicebio, China). The samples were then refrigerated overnight at 4°C. The next day, the slices were cleaned three times, and the secondary antibody Fitc (G1222, Servicebio, China) or Cy3 (GB123, Servicebio, China) was incubated at room temperature in the dark for 1 h. DAPI was used to stain the tissues’ nuclei, and the samples were cleaned and sealed with a cover glass. A fluorescence microscope was used to observe and collect fluorescence images. The fluorescence intensity was analyzed by ImageJ software (Meng et al., 2016).

### Statistical Analysis

All results are expressed as the mean ± standard deviation (SD). GraphPad Prism software (GraphPad Software Inc., San Diego, CA, USA) was used for data analysis. All data were tested for normal distribution and homogeneity of variance. Unpaired *t*-test was used in the comparison between the two groups, and one-way ANOVA analysis followed by Tukey’s multiple comparisons test was used in the multi-group pairwise comparison. A *p*-value <0.05 was considered to be statistically significant.

## Results

### The Learning and Memory Abilities of AD Model Mice

**Figure 1:**
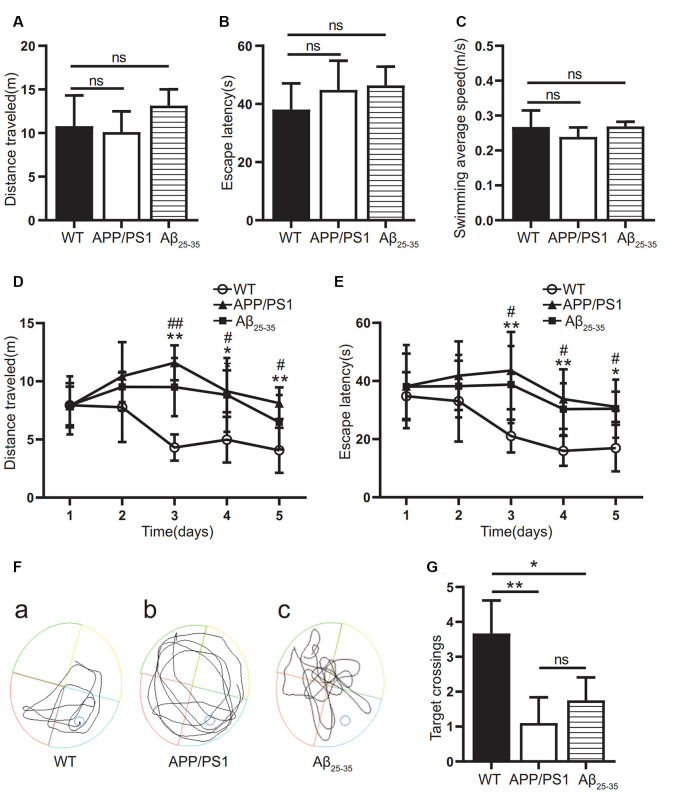
Cognitive function of Alzheimer’s disease (AD) model mice. **(A–C)** The swimming distance, incubation period, and average swimming speed of the APP/PS1 group and Aβ_25–35_ group mice were studied in the Morris water maze (MWM) experiment with a visual platform. **(D,E)** The swimming distance and latency of the APP/PS1 group and the Aβ_25–35_ group in the MWM experiment with a hidden platform (WT vs. APP/PS1 group: *p < 0.05, **p < 0.01; WT vs. Aβ_25–35_ group: ^#^*p* < 0.05, ^##^*p* < 0.01; *n* = 10). **(F)** Swimming path map of the APP/PS1 group and Aβ_25–35_ group mice in the water tank. **(G)** The number of platform crossings of the Aβ_25–35_ group and APP/PS1 group mice after removing the platform (*p < 0.05, **p < 0.01; “ns” means there is no significant difference; *n* = 10).

In this study, the WMW test was used to verify whether the cognitive function was impaired in the APP/PS1 group and the Aβ_25–35_ group. On the first day, the swimming distance, incubation period, and average swimming speed before reaching the platform of the mice in each group were tested by a visual platform (Figures 1A–C). The results showed that there was no significant difference among the groups (*p* > 0.05). After the platform was hidden, we assessed the ability of the mice to find and memorize the platform position. During the 5-day experiment, the swimming distance and latency of the mice in each group showed a downward trend (Figures 1D–F). The swimming distance and latency of the APP/PS1 and Aβ_25–35_ groups were longer than those of the WT group (Figures 1D,E). On the 7th day, after the platform was removed, the number of times the mice crossed the original platform was detected to test the memory retention ability. The results showed that the number of platform crossings in the APP/PS1 group and the Aβ_25–35_ group was lower than that in the WT group (*p* < 0.05; Figure 1G). The results showed that both the APP/PS1 group and the Aβ_25–35_ group had cognitive impairment.

### The Levels of Inflammatory-Related Proteins and Inflammatory Factors in the Brain Tissue of AD Model Mice Increased

**Figure 2:**
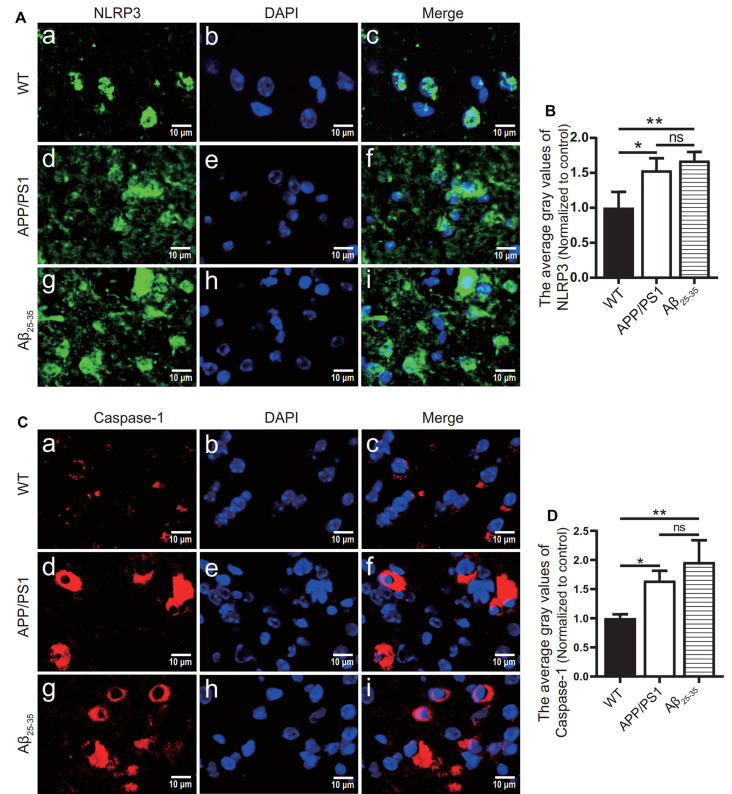
NLRP3 inflammasome-related proteins were increased in AD model mice. **(A,C)** Immunofluorescence was used to analyze the expression of the NLRP3 inflammasome-related proteins NLRP3 and caspase-1 in the brain tissues of the WT group, APP/PS1 group, and Aβ_25–35_ group mice. **(B,D)** The difference in fluorescence intensity between groups was compared (*p < 0.05, **p < 0.01; “ns” means there is no significant difference; *n* = 3).

**Figure 3:**
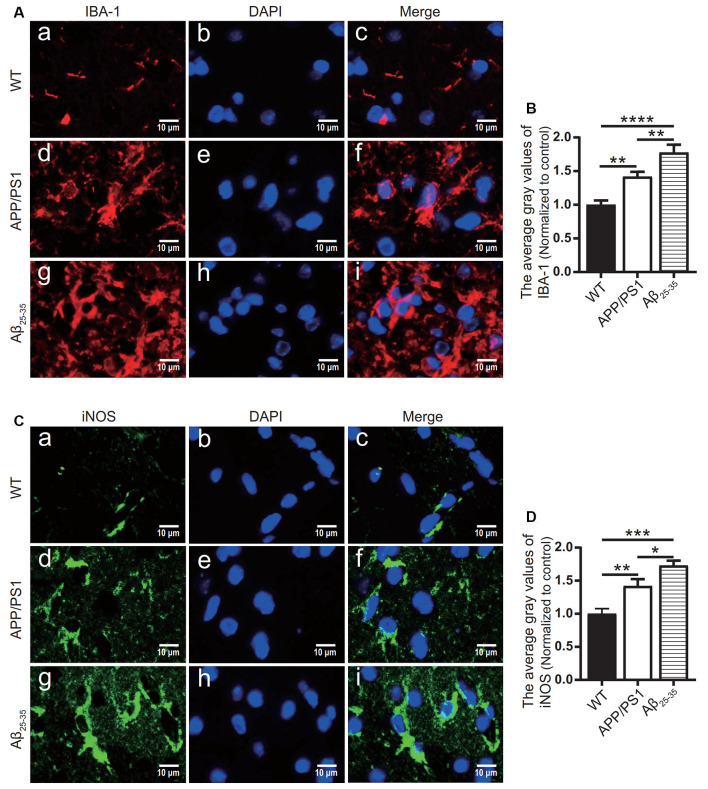
Microglial marker proteins and inflammatory proteins were increased in AD model mice. **(A,C)** Immunofluorescence was used to analyze the microglial marker protein IBA-1 and inflammatory protein iNOS in the brain tissues of the WT group, APP/PS1 group, and Aβ_25–35_ group mice. **(B,D)** The difference in fluorescence intensity between groups was compared (*p < 0.05, **p < 0.01, ***p < 0.001, ****p < 0.0001; *n* = 3).

**Figure 4:**
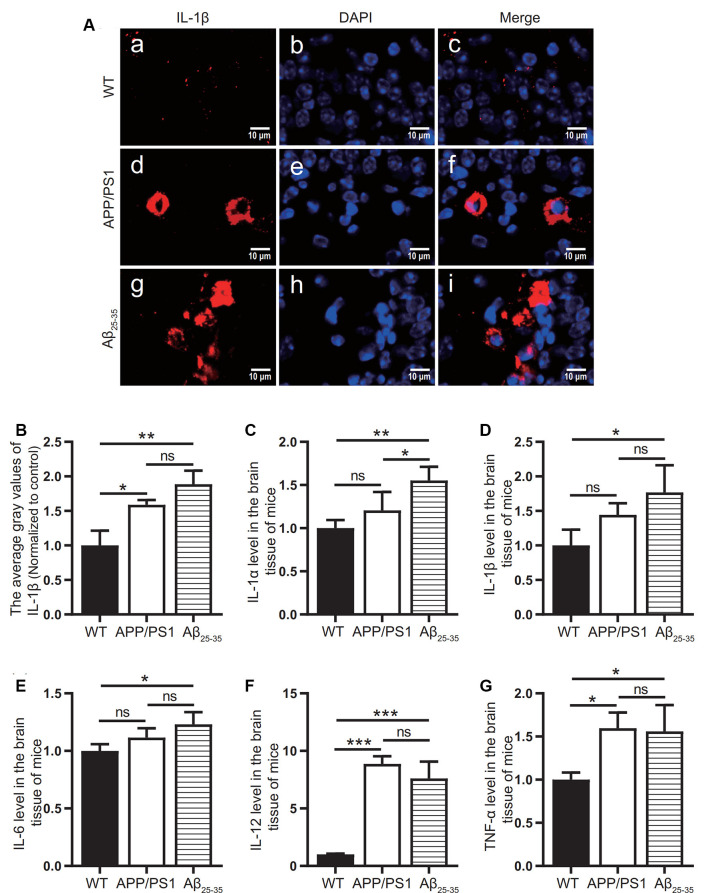
Expression of inflammatory factors in AD animal models. **(A)** Immunofluorescence was used to analyze the inflammatory protein IL-1β in the brain tissues of the WT group, APP/PS1 group, and Aβ_25–35_ group. **(B)** The difference in fluorescence intensity between groups was compared. **(C–G)** The expression levels of the inflammatory factors IL-1α, IL-1β, IL-6, IL-12, and TNF-α in the brain tissues of the WT group, APP/PS1 group, and Aβ_25–35_ group mice were detected (*p < 0.05, **p < 0.01, ***p < 0.001; “ns” means there is no significant difference; *n* = 3).

To observe the activation of the inflammatory response in the AD animal model, we detected inflammatory proteins, microglial markers, and inflammatory factors in the brain tissue of the APP/PS1 group mice and Aβ_25–35_ group mice. The expression of the NLRP3 inflammasome-related proteins NLRP3 (Figures 2A,B), caspase-1 (Figures 2C,D), and IL-1β (Figures 4A,B) in the brain tissues of the APP/PS1 group and Aβ_25–35_ group mice was significantly higher than that in the brain tissues of the control group mice (*p* < 0.05). The expression of IBA-1 protein (Figures 3A,B) was significantly higher than that in the control group (*p* < 0.05). The level of iNOS protein (Figures 3C,D) in M1 microglia polarization was also notably higher than that in the control group (*p* < 0.05). These results suggest that the NLRP3 inflammasome and microglia in the brain tissue of AD model mice are activated.

The levels of IL-1α, IL-1β, IL-6, IL-12, and TNF-α in the brain tissue of mice in the Aβ_25–35_ group were significantly increased (*p* < 0.05; Figures 4C–G). In the APP/PS1 group, the levels of the inflammatory factors IL-12 and TNF-α in the brain tissue were increased (*p* < 0.05; Figures 4A,B), while the levels of the inflammatory factors IL-1α, IL-1β, and IL-6 were not significantly increased (*p* > 0.05; Figures 4C–E). The above results showed that the immune response of the AD model mice was activated.

### Activation of NLRP3 Inflammasome in AD Cell Models

**Figure 5:**
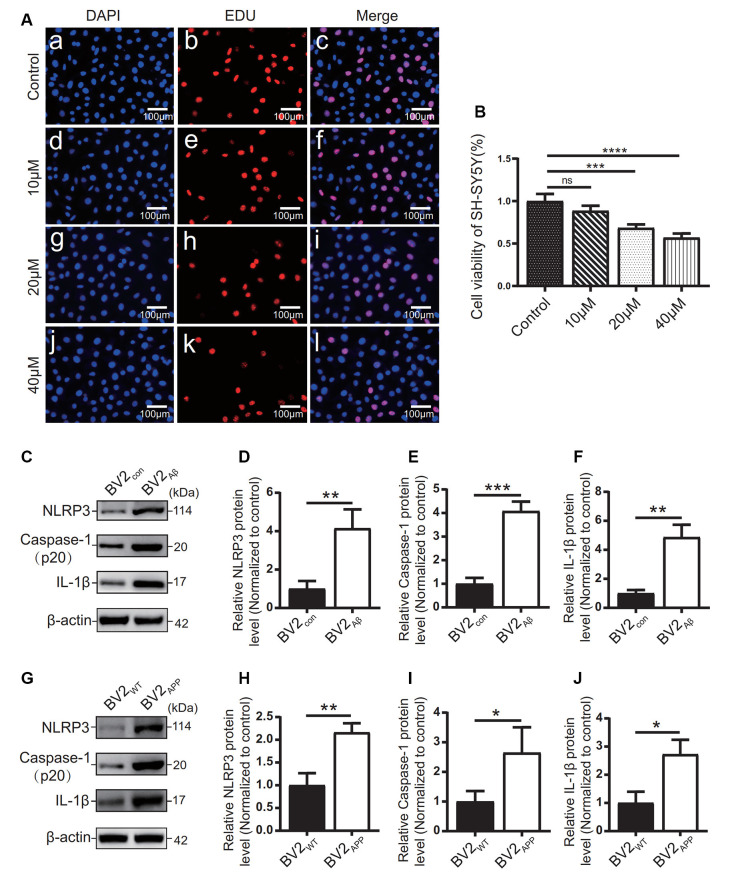
Influence of Aβ_25–35_ on SH-SY5Y and expression of inflammatory proteins in AD cell models. **(A)** DAPI and EdU staining were used to observe the number of nuclei in the SH-SY5Y cells treated with 10, 20, and 40 μM Aβ_25–35_ for 24 h, and **(B)** the proliferation ratio of each group was calculated. BV2 cells were cocultured with SH-SY5Y cells or SH-SY5Y cells supplemented with Aβ_25–35_ for 24 h. **(C)** The expression of the NLRP3, caspase-1, and IL-1β proteins in BV2 cells was detected by Western blots. **(D–F)** The relative gray value of protein expression in each group was compared. After BV2 cells were cocultured with APP_WT_ or APPswe cells. **(G)** Western blotting was used to detect the expression of NLRP3, caspase-1, and IL-1β. **(H–J)** The relative gray values of NLRP3, caspase-1, and IL-1β in each group (*p < 0.05, **p < 0.01, ***p < 0.001, ****p < 0.0001; “ns” means there is no significant difference; *n* = 3).

The effects of different concentrations of Aβ_25–35_ on SH-SY5Y cells were observed by EdU staining (Figures 5A,B). The results showed that 10 μM Aβ_25–35_ had no significant effect on the proliferation rate of SH-SY5Y cells (Figure 5B). 20 μM and 40 μM Aβ_25–35_ caused a gradual decrease in the proliferation rate of SH-SY5Y cells (*p* < 0.05; Figure 5B). Therefore, at 20 μM, the proliferation rate of SH-SY5Y cells decreased with increasing Aβ_25–35_ concentration.

The expression levels of inflammatory-related proteins in BV2 cells cocultured with the APPswe group or Aβ_25–35_ group cells were detected by Western blots. The results showed that the protein levels of NLRP3, caspase-1, and IL-1β in the BV2_APP_ group or BV2_Aβ_ group were higher than those in the control group (*p* < 0.05; Figures 5C–J).

### AD Model Mice and Cells Showed Abnormal Autophagic Degradation

**Figure 6:**
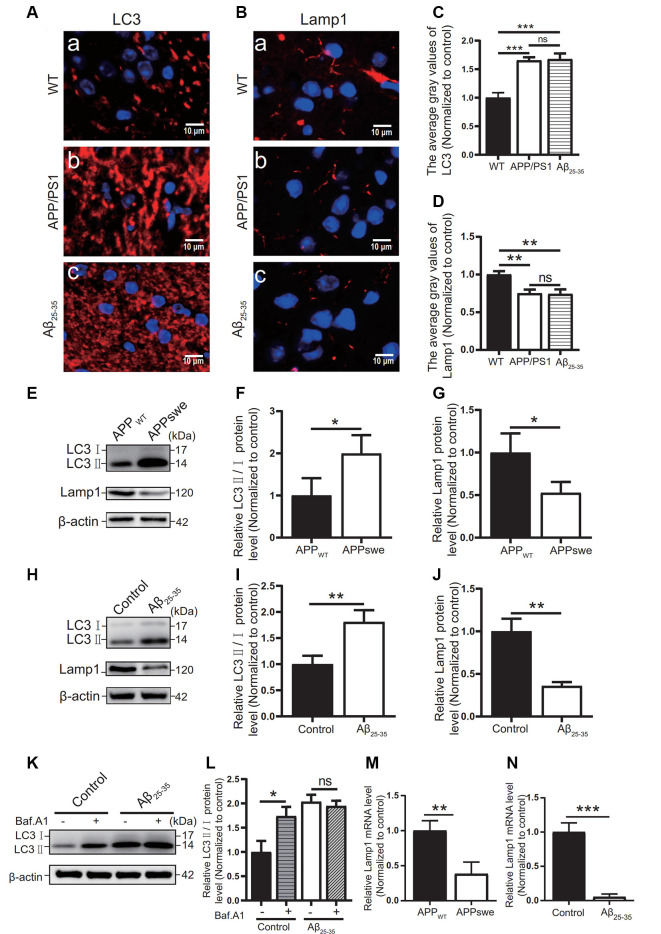
Abnormal autophagic degradation in AD animal models and cell models. Immunofluorescence was used to observe the expression of the LC3 and Lamp1 proteins in the WT group, APP/PS1 group, and Aβ_25–35_ group mice; the red fluorescence shows the immunofluorescence staining of LC3 **(A,C)** or Lamp1 **(B,D)** protein. **(E)** Western blot showing the expression of the LC3 and Lamp1 proteins in the APP_WT_ group and APPswe group cells, **(F,G)** and the relative gray value of the protein. **(H)** Western blot showing the protein expression of LC3 and Lamp1 in the control group and Aβ_25–35_ group cells. **(I,J)** The relative gray value of the protein. **(K)** Western blot showing LC3 protein expression in the control group and Aβ_25–35_ group cells before and after adding bafilomycin A1, and **(L)** the relative gray value of each group was compared. **(M,N)** PCR quantitatively analyzed the mRNA expression of Lamp1 in the APPswe group cells or Aβ_25–35_ group cells (*p < 0.05, **p < 0.01, ***p < 0.001; “ns” means there is no significant difference; *n* = 3).

The autophagic function of AD animal and cell models was further observed. Immunofluorescence tests showed that the LC3 protein expression (Figures 6A,C) in the APP/PS1 group mice and the Aβ_25–35_ group mice was higher than that of the WT group (*p* < 0.05).

In Western blot experiments, the LC3 protein expression in the APPswe group cells and Aβ_25–35_ group cells was compared with that of the control group. The results showed that the ratio of LC3II/I in the APPswe group cells and the Aβ_25–35_ group cells was increased (*p* < 0.05; Figures 6E,F,H,I).

To distinguish whether the LC3 level increase is due to early autophagic activation or late autophagic inhibition, we performed LC3 turnover assays (Figures 6K,L) of the Aβ_25–35_ group cells. Western blotting was used to observe the LC3 protein levels in the control group and Aβ_25–35_ group cells before and after adding bafilomycin A1. The results showed that the LC3II/I protein ratio in the control group cells increased after adding bafilomycin A1 (*p* < 0.05). The ratio of LC3II/I in the Aβ_25–35_ group cells was not significantly increased after adding bafilomycin A1 (*p* > 0.05). These results indicated that AD animal and cell models may show abnormal autophagic degradation.

Moreover, the expression of Lamp1 protein was detected in different ways. Immunofluorescence assays showed that Lamp1 protein expression in both AD model mice was decreased compared with that in the WT group mice (*p* < 0.05; **Figures 6B,D**). The Lamp1 protein expression was decreased in the APPswe group cells and Aβ_25–35_ group cells, as detected by Western blots (*p* < 0.05; Figures 6E,G,H,J). The expression of Lamp1 nucleic acid was detected by PCR assays, and we found that Lamp1 nucleic acid levels in the APPswe group cells and Aβ_25–35_ group cells were significantly lower than those in the control group (*p* < 0.05; Figures 6M,N). These results suggest that abnormal autophagic degradation in AD animal and cell models may be related to a decrease in lysosomal function.

### Overexpression of TFEB in an AD Coculture Cell Model Can Alleviate the Inflammatory Reaction

**Figure 7:**
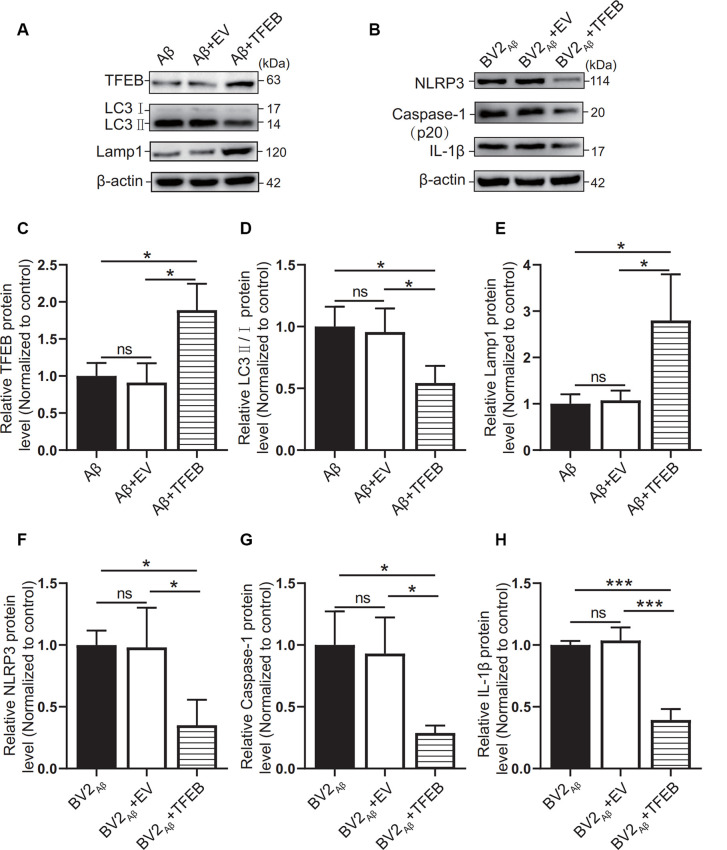
Effects of TFEB overexpression on autophagy in SH-SY5Y cells supplemented with Aβ_25–35_. After overexpression of the TFEB protein in the control group or Aβ_25–35_ group cocultured with BV2, **(A)** the expression of the TFEB, LC3, and Lamp1 proteins in the Aβ group, the Aβ + EV group, and the Aβ + TFEB overexpression adenovirus group was observed by Western blots, and **(C–E)** the relative gray values of each group were compared.** (B)** Western blotting was used to observe NLRP3, caspase-1, and IL-1β protein expression in BV2 cells, and **(F–H)** the relative gray values of each group were compared (*p < 0.05, ***p < 0.001; “ns” means there is no significant difference; *n* = 3).

To observe the autophagic and lysosomal function of the Aβ_25–35_ group cells and the inflammatory response of BV2 cells, TFEB protein overexpression adenovirus was added to the coculture model of BV2 and SH-SY5Y cells supplemented with Aβ_25–35_. The results showed that TFEB protein expression was increased in the Aβ + TFEB group cells overexpressing TFEB (*p* < 0.05; Figures 7A,C). The LC3 protein level was reduced (*p* < 0.05; Figures 7A,D). The level of the lysosomal membrane protein Lamp1 was increased (*p* < 0.05; Figures 7A,E). The protein expression of NLRP3, caspase-1, and IL-1β in the BV2 cells overexpressing TFEB was reduced (*p* < 0.05; Figures 7B,F–H). The results showed that overexpression of the TFEB protein might improve autophagic function by enhancing lysosomal function and alleviating inflammatory reactions.

## Discussion

Neuroinflammation is a pathological marker for AD progression (Zhang et al., 2016) and plays an important role in the occurrence and development of AD (Song et al., 2017). The NLRP3 inflammasome is an inflammatory body that has attracted increasing attention in recent years. After assembly and activation by NLRP3, ASC, and caspase-1 protein, pro-caspase-1 can be transformed into active caspase-1. Then, pro-il-1β and pro-il-18 are sheared to form IL-1β and IL-18 with inflammatory activity (Wree et al., 2013). The inflammatory response caused by overactivated NLRP3 inflammasomes can lead to neuronal damage and organ dysfunction (Kim et al., 2016; You et al., 2017).

Autophagy plays a beneficial role in scavenging abnormal proteins, especially Aβ and tau proteins (Hamano et al., 2018). LC3 and p62 are important proteins in the formation of autophagy (Palmieri et al., 2011). P62 binds LC3 and recruits abnormal proteins or damaged organelles for autophagy (Wang et al., 2019). During autophagic and lysosomal degradation of autophagic substrates, the lysosomal-related membrane protein Lamp1 plays an important role in regulating lysosomal activity, which is conducive to the formation of autolysosomes (Eskelinen et al., 2004; Huynh et al., 2007). This process is essential to decompose autophagic substrates with the help of abundant proteases in the lysosome (Kim et al., 2016).

Therefore, autophagy and inflammation play an essential role in the pathogenesis of AD (Harris et al., 2017; Fang et al., 2018; BostancıKlıoğlu, 2019), but the relationship between them is still unclear. In this study, An AD model generated by overexpression of the APP gene and exogenous Aβ_25–35_ was used to explore the possible relationship between immune-inflammatory activation and autophagy.

APP/PS1 mice were injected with exogenous Aβ_25–35_ *via* the lateral ventricles for the AD animal model (Fan et al., 2015; Gong et al., 2019). For the AD cell model, APPswe cells stably transfected with the APP gene, and SH-SY5Y cells induced by exogenous Aβ_25–35_ were used. To explore the effect of different concentrations of Aβ_25–35_ solution on SH-SY5Y cells, we carried out EdU experiments and found that 20 μM was the minimum dose to reduce the proliferative activity of SH-SY5Y cells. Therefore, in the AD cell models constructed with Aβ_25–35_, SH-SY5Y cells were treated with 20 μM Aβ_25–35_ for 24 h. This modeling parameter is also consistent with previous research methods (Kaur et al., 2015). Also, to construct a cell environment as close as possible to AD lesions *in vivo*, we cocultured the AD cell models with BV2 and APPswe or SH-SY5Y cells supplemented with Aβ_25–35_.

AD is a disease with cognitive impairment as its clinical manifestation. In experiments with AD animal models, the MWM assay is commonly used to detect the learning ability and memory of the animals (Baeta-Corral and Giménez-Llort, 2015; Gong et al., 2019; Sun et al., 2019). In this study, APP/PS1 double-transgenic mice and Aβ_25–35_-injected mice were subjected to the MWM test, and they had obvious cognitive dysfunction, consistent with the behavioral phenotype of the AD animal model (Tian et al., 2019).

In this study, NLRP3, caspase-1, and IL-1β were increased in the brain tissues of the AD model mice, which was consistent with previous research results (François et al., 2014). In *in vitro* experiments, NLRP3, caspase-1, and IL-1β were increased in BV2 cells. At the level of inflammatory factors, IL-1α, IL-1β, IL-6, IL-12, and TNF-α in the brain tissues of the Aβ_25–35_ group mice were increased, while only IL-12 and TNF-α levels in APP/PS1 group were increased. Also, the levels of IBA-1 and iNOS were increased in the AD animal models. IBA-1 is a marker used to detect microglia, and its high expression level indicates an increase in microglia (Zhao et al., 2019). iNOS is an important inflammatory signal molecule. The increase in iNOS also indicates that microglia are activated by inflammatory stimulation (Issy et al., 2018). Therefore, AD animal models have different degrees of inflammatory activation, and NLRP3 inflammatory bodies may mediate this inflammatory reaction. Compared with the WT group, the APP/PS1 group and the APPswe group also had activation of the inflammatory response, but the degree of the inflammatory reaction was not as strong as that of the AD animals and cells induced by exogenous Aβ_25–35_.

Autophagy shows dynamic changes in the development of AD, and the role of autophagy in inflammation remains controversial. Many studies have shown that AD pathogenesis is accompanied by autophagic disorder (Peric and Annaert, 2015; Zhang et al., 2016), which may occur in different stages of autophagic flux. In our previous study, we explored the function of autophagic flux in AD (Long et al., 2020). Immunofluorescence staining showed that autophagosomes accumulated in the brain tissues of AD patients, and the lysosomal marker Lamp1 was decreased. In AD animal and cell models generated by APP overexpression or exogenous Aβ, the expression of autophagic markers in different stages of autophagic flux was observed, and we found that the level of Lamp1 was decreased in the AD animal and cell models. We speculate that the abnormal lysosomal function may be an essential cause of autophagic dysfunction. Based on our previous studies, this study further explored the dysfunction of autophagic flux to examine the key link between autophagic disorders and the inflammatory response in AD.

In this study, immunofluorescence and Western blotting were utilized to detect the protein expression of LC3 in AD animals and cell models, respectively. We found that the protein expression level of LC3 was increased. This finding is consistent with previous studies (François et al., 2015; Alvarez-Arellano et al., 2018). LC3 is a universal biochemical index for observing autophagy and assessing autophagic activity. Previous studies generally considered that the LC3 increase is due to autophagic activation (Rodgers et al., 2014). However, in recent years, some scholars have questioned whether the increase in LC3 levels is caused by autophagic activation or the degradation of autophagic products, and this question can be addressed by the LC3 turnover test (Yoshii and Mizushima, 2017). According to Yoshii’s research method, if early autophagic activation leads to an increase in LC3 levels, early autophagy will not be affected after bafilomycin A1 addition in late autophagy, and LC3 will continue to increase due to the inhibition of degradation. If LC3 protein accumulation is caused by late autophagic inhibition, the LC3 level will not be affected if bafilomycin A1 is used to inhibit late autophagy (Yoshii and Mizushima, 2017). Our results showed that the LC3 level in the Aβ_25–35_ group cells did not further increase after adding bafilomycin A1 to inhibit late autophagy, suggesting that there was abnormal late autophagy. Furthermore, we found that the protein expression of Lamp1 in the AD animal and cell models and the nucleic acid expression of Lamp1 in the AD cell models were decreased. These results suggest that lysosomal function may be impaired in the AD cell model and suggest that autophagic dysfunction may be caused by lysosomal dysfunction in the AD model. Because autophagy is impaired, toxic proteins cannot be normally degraded, which may cause secondary inflammatory reactions (Alvarez-Arellano et al., 2018).

Activation of the NLRP3-mediated inflammatory response may be caused by abnormal autophagic degradation. Aβ can activate the NLRP3 inflammasome, which has been confirmed by many studies (Zhang et al., 2016; Ahmed et al., 2017; You et al., 2017). The levels of SAPα and Aβ_1–40_ in the APPswe medium were increased, which confirmed that APPswe cells secreted extracellular APP and Aβ (Fernandes et al., 2018). Under normal conditions, Aβ produced by nerve cells can be cleared by autophagy. However, dysfunctional autophagy leads to Aβ accumulation. Toxic Aβ may activate NLRP3 inflammasome-mediated inflammation through Nod-like receptors or Toll-like receptors (You et al., 2017; Alvarez-Arellano et al., 2018). The accumulation of misfolded proteins can induce lysosomal damage, which not only further reduces the degradation of autophagic-lysosomal substrates, resulting in a high level of autophagosome-lysosome accumulation (Yang et al., 2014), but also leads to changes in lysosomal membrane permeability, resulting in a substantial release of Cathepsin B, thus activating NLRP3 inflammatory body activation (Li et al., 2013; Shin et al., 2013; Pomilio et al., 2020). Also, decreased autophagy may result in the accumulation of ROS produced by damaged mitochondria and activate NLRP3 inflammatory bodies (Harris et al., 2017; Sun et al., 2017). Studies have further shown that autophagy-related proteins such as p62 (Sun et al., 2017) and Beclin-1 (Salminen et al., 2013) directly interact with inflammatory pathways during the interaction between autophagy and inflammation.

Therefore, abnormal autophagic degradation may be involved in the activation of the NLRP3 inflammasome in various ways (Kim et al., 2016). After activation of the NLRP3 inflammasome, the secretion of inflammatory cytokines such as IL-1β and TNF induced by NLRP3 leads to a strong inflammatory reaction, impairs nerve cell function, and finally leads to cognitive dysfunction (Nurmi et al., 2017; Alvarez-Arellano et al., 2018).

Since autophagy is closely related to inflammation, can inflammation be improved by enhancing lysosomal function in autophagic flux? Some studies have shown that in cells overexpressing TFEB, the number of lysosomes increases, and the degradation of lysosomal substrates and autophagic substrates is enhanced (Sardiello et al., 2009). Xiao’s study showed that TFEB overexpression in primary astrocytes enhanced the degradation of Aβ_42_, and senile plaques in the hippocampus of APP/PS1 mice overexpressing TFEB decreased by 45% compared with those of the control group (Xiao et al., 2014). Yamamoto’s study found that TFEB overexpression upregulated the expression of LAMP-1 (Yamamoto et al., 2019). Additionally, in the classification of differentially expressed genes targeting TFEB, it was found that lysosome-related genes were the most enriched, and they also interacted with autophagy-related genes such as Beclin-1 (Palmieri et al., 2011). These findings indicate that TFEB is closely related to lysosomes and autophagy. TFEB may enhance the degradation of autophagic substrates by improving lysosomal function.

In this study, TFEB-overexpressing adenovirus was used to treat the Aβ_25–35_ group cells cocultured with BV2 cells. The level of the autophagic marker LC3 was decreased, and the level of the lysosomal membrane protein Lamp1 was increased in the Aβ + TFEB group cells cocultured with BV2 cells. The expression levels of NLRP3, caspase-1, and IL-1β in BV2 cells were also decreased. The mechanism may be related to TFEB protein indirectly improving late autophagy. TFEB enhanced lysosomal activity, increased the degradation of accumulated autophagic lysosomes, and promoted autophagic flux patency. When autophagic flux is unobstructed, the accumulation of upstream substrates or downstream autophagic products will decrease, which will decrease the activation of inflammatory cells by toxic products.

## Conclusion

In conclusion, the results of this study suggest that the NLRP3 inflammasome-mediated immune inflammatory response is activated in AD animal models and cell models, which may be related to the dysfunction of autophagic degradation caused by the decline in lysosomal function. After the TFEB protein was overexpressed in the AD cell model, NLRP3 inflammasome-related proteins in BV2 cells decreased. This mechanism may be associated with the recovery of lysosomal function and the improvement of late autophagic function, which indirectly alleviated the inflammatory reaction.

## Data Availability Statement

The raw data from the current study are available from the corresponding authors.

## Ethics Statement

The animal study was reviewed and approved by Ethics Committee of Chongqing Medical University.

## Author Contributions

GH, LY, ZL, and WZ conceived, designed, and supervised the experiments. WZ and ZL performed most of the experiments and analyzed the data. DX, YZ, and BT participated in the animal experiments and cell experiments and acquired data. WZ drafted the manuscript and graphs. ZL and GH reviewed and edited the manuscript and graphs. All authors have read and approved the final manuscript.

## Conflict of Interest

The authors declare that the research was conducted in the absence of any commercial or financial relationships that could be construed as a potential conflict of interest.

## Abbreviations

AD: Alzheimer’s disease
NLRP3: NOD-like receptor family pyrin domain containing 3
MWM: Morris water maze
APP/PS1: Swedish mutant form of APP (APPswe)/PSEN1dE9 transgenic mice
Aβ_25–35_: amyloid-β 25–35
IL-1α: interleukin-1α
IL-1β: interleukin-1β
IL-6: interleukin-6
IL-12: interleukin-12
TNF-α: tumor necrosis factor-α
IBA-1: ionized calcium-binding adapter molecule 1
iNOS: inducible nitric oxide synthase
APPswe: Swedish APP mutant
LC3: microtubule-associated protein 1A/1B light chain-3
Lamp1: lysosomal-associated membrane protein 1
TFEB: transcription factor EB.
